# Is There an Ethical Dilemma in Xenotransplantation?

**DOI:** 10.1111/xen.70135

**Published:** 2026-04-29

**Authors:** Joachim Denner

**Affiliations:** ^1^ Institute For Virology Free University Berlin Berlin Germany

**Keywords:** infection, lifelong surveillance, Ulysses contract, withdrawal, xenotransplantation

## Abstract

As clinical trials in xenotransplantation commence, the question of whether participants may withdraw from research is being intensely debated. Some authors argue that xenotransplant recipients should be subject to lifelong monitoring because of the potential risk of zoonotic or xenozoonotic infections affecting third parties. Others maintain that the right to withdraw from research participation is a fundamental principle of medical ethics that must not be compromised. To clarify this tension, historical precedents have been examined, and the applicability of so‐called Ulysses contracts to xenotransplantation has been critically assessed. The practical answer is more straightforward than the theoretical debate suggests: xenotransplant recipients, like allotransplant recipients, require continuous medical supervision. Lifelong immunosuppression and regular follow‐up are essential to preserve transplant function and, ultimately, the patient's life.

## 1 Introduction

The ethical debate on xenotransplantation has recently focused on a central dilemma: how to balance the right of transplant recipients to withdraw from research participation with the perceived need for mandatory lifelong surveillance to protect public health [1]. Entwistle et al. and Hawthorne [2, 3] argue that xenotransplant recipients should be subject to long‐term, potentially lifelong, monitoring because of possible risks from zoonotic or xenozoonotic infections for third‐parties. In their view, uncertainty about the presence, latency, and transmissibility of porcine viruses justifies stringent and non‐waivable follow‐up requirements.

Bobier and co‐authors [4] have criticized this position, particularly because the right to withdraw from research participation is regarded as a fundamental principle of medical ethics. They have framed this issue as an ethical conflict between individual autonomy and collective safety [5]. They present illustrative scenarios in which a xenotransplant recipient develops an infection caused by a porcine virus and, after withdrawing from monitoring, inadvertently exposes others before the infection is recognized. These examples are meant to demonstrate that allowing recipients to opt out of surveillance could endanger the public and therefore raise questions about whether the traditional right to withdraw from research should apply in xenotransplantation.

## 2 Current State of Practice

However, when considered in light of the clinical realities of transplantation, this dilemma largely dissolves. Both, allotransplantation and xenotransplantation require lifelong immunosuppressive therapy to prevent transplant rejection. Continuous medical supervision is therefore not optional but inherent to posttransplant survival: discontinuation of follow‐up or medication would inevitably lead to transplant loss and death. As a result, transplant recipients cannot realistically disengage from medical care, regardless of whether the graft originates from a human or a pig. In routine clinical practice, informed consent for organ transplantation already includes the requirement of lifelong follow‐up at a transplant center. There is no practical reason why xenotransplantation should differ in this regard.

The debate about mandatory surveillance must also be understood against the background of the potential virological risks associated with xenotransplantation. Encouraged by preclinical studies transplanting pig islet cells, kidneys, livers, and hearts into nonhuman primates with increasingly long survival times [6], initial clinical xenotransplantations have been undertaken, including transplantations of pig organs into brain‐dead recipients and living patients [7 ‐10]. While early heart xenotransplants resulted in survival of only several weeks, pig kidney xenotransplants have already achieved survival of months.

Nevertheless, infectious risks remain a concern [11]. Porcine viruses can influence transplant survival and may be transmitted with the donor organ. For example, transmission of porcine cytomegalovirus, which is a porcine roseolovirus (PCMV/PRV), has been shown to markedly reduce survival in nonhuman primate recipients of pig hearts or kidneys [12, 13, 14]. In the first clinical pig‐heart xenotransplantation, PCMV/PRV was transmitted with the donor organ and contributed to the recipient's death [9]. Importantly, however, this virus does not infect human cells [15] and does not cause disease in humans outside the xenotransplant setting. Instead, it replicates within the transplanted organ and appears to affect the recipient indirectly through interactions with immune and endothelial cells [16]. For this reason, it is better characterized as xenozoonotic rather than zoonotic.

These observations underscore the importance of rigorous donor screening, sensitive detection methods, and strategies to eliminate porcine viruses prior to transplantation. Advances in screening donor pigs make it increasingly feasible to provide organs that are virologically cleaner than many human donor organs. Indeed, allotransplantation has historically involved transmission of numerous human pathogens including cytomegalovirus, Epstein–Barr virus, hepatitis viruses, rabies virus and others [17]. Xenotransplantation may ultimately offer advantages in this respect because donor animals can be raised under controlled conditions and repeatedly tested.

In real‐world transplantation practice, recipients are closely monitored for life and remain highly motivated to maintain contact with their transplant teams. Any symptoms of an illness would prompt immediate evaluation in a specialized transplant center, where screening for both human and porcine pathogens could be rapidly initiated. Potential contacts could then be traced and assessed if necessary. The hypothetical scenario of a recipient abandoning follow‐up and unknowingly spreading infection is therefore highly implausible in clinical reality, as ongoing medical care is indispensable for survival.

Bobier, Hurst and others [4, 5, 18] ask that if the risk to public health is so great as to say that xenograft recipients must submit to lifelong monitoring for infectious diseases then perhaps we're not ready for xenotransplantation. However, we are ready for xenotransplantation. In recent years, enormous progress has been made in the field of virological safety of xenotransplantation, including improved detection methods for potentially pathogenic viruses as well as strategies to prevent virus transmission [19, 20, 21, 22]. The fact that a porcine herpesvirus, porcine cytomegalovirus/porcine roseolovirus (PCMV/PRV), was transmitted to the first patient receiving a pig heart was due to the use of an inappropriate screening method, despite the fact that the correct methods had been available since 2016 [23, 24].

## 3 Historical Precedents

Looking back at the historical foundations that define the limits of permissible medical experimentation on human subjects, the Nuremberg Code states in its first principle: “The voluntary consent of the human subject is absolutely essential.” [25]. Although xenotransplantation today remains in an experimental phase, it is likely to enter clinical practice in the foreseeable future. However, the Code was drafted in the aftermath of the atrocities committed in German concentration camps, and its primary aim was to prevent coercive and abusive experimentation. In 1946 –1947, no one anticipated biomedical developments that would involve not only complex transplantation procedures, but also lifelong immunosuppression and long‐term public health monitoring.

A similar limitation is inherent in the Guidance on Withdrawal of Subjects from Research issued by the U.S. Department of Health and Human Services (HHS) under 45 CFR 46.116(a)(8) [26]. This guidance clearly states: “Subjects have the right to withdraw from (i.e., discontinue participation in) research at any time (45 CFR 46.116(a) (8)).” It further specifies that if a subject decides to withdraw from all components of a research study, the investigator must discontinue all research activities involving that subject's participation in the study, in accordance with 45 CFR 46.116(a)(8). “Interacting or intervening with the subject in order to obtain data about him or her for the research study (e.g., administering an experimental drug, performing a tissue biopsy, drawing blood, exposing the subject to visual stimuli on a computer monitor and measuring response times, orchestrating environmental events or social interactions, or conducting ethnographic interviews with the subject)” [26].

However, this guidance was developed primarily with conventional clinical trials in mind, such as studies of new drugs or vaccines, not with procedures like xenotransplantation. Its framework presupposes research participation that can be discontinued without necessarily creating ongoing medical dependency or long‐term public health implications. Importantly, the same applies to allotransplantation. Solid‐organ transplantation likewise requires lifelong immunosuppression, and severe immunosuppression may lead to the reactivation of latent human viruses or increase susceptibility to viral infections from the transplant or the environment that can replicate in the host. In such contexts, the simple model of withdrawal does not adequately capture the medical and ethical realities of permanent transplant dependence and continuous monitoring.

For this reason, it would be desirable for future revisions of this guidance to explicitly address the special situation of xenotransplantation and comparable cellular or organ‐based therapies that entail lifelong treatment and long‐term monitoring. Such clarification would help reconcile the principle of withdrawal with the unique clinical and public health obligations inherent in these forms of therapy.

Closer to the practical situation is the FDA guidance for xenotransplantation which states: “The informed consent document should contain information about the proposed lifelong surveillance for all recipients and the need for clinical and laboratory monitoring throughout. You should explain, to the extent possible, the schedule for such clinical and laboratory monitoring” [27]. This implies that the patient has to be informed that he has to take lifelong immunosuppressiva otherwise the transplant will be rejected and that this is associated automatically with a follow up of his health status.

## 4 Ulysses Contract

Several authors have proposed applying the concept of a Ulysses contract to xenotransplantation [28, 29]. For example, Martine Rothblatt discusses the first cardiac xenotransplantation and describes Ulysses contracts as a “kind of non‐withdrawable informed consents to comply with infection control procedures.” [30].

This type of contract takes its name from Homer's epic poem The Odyssey [31], in which the hero Odysseus (known in Latin as Ulysses or Ulixes) wishes to hear the enchanting yet perilous song of the Sirens without endangering his ship. Following the advice of the goddess Circe, he orders his crew to plug their ears with wax and to bind him tightly to the mast. Aware that he might later beg to be released, Ulysses makes a prior agreement with his sailors that they must not untie him until they have safely passed beyond the Sirens’ reach.

Ulysses contracts were introduced first in the field of psychiatry as psychiatric advance directives (PAD) and cannot be applied to xenotransplantation. A Ulysses contract is typically justified when a person anticipates a future period of impaired decision‐making (e.g., during psychiatric relapse) and voluntarily authorizes others to override their later refusals. In xenotransplantation, however, there is no episode of temporary irrationality that would justify advance self‐binding. Recipients are not anticipating a future period of impaired decision‐making; rather, they are entering into a form of treatment that entails ongoing medical obligations. Hurst et al. [32] also argued that the application of Ulysses contracts to xenotransplantation is inappropriate. Xenotransplantation protocols require lifelong surveillance, not only because of the potential risk of zoonotic infection, but primarily due to the need for continuous immunosuppression. Without sustained immunosuppressive therapy, the transplant will be rejected and the patient will die, just as in allotransplantation. In the course of allotransplantation also no Ulysses contract is needed. Informed consent for allotransplantation routinely includes the statement that “lifelong aftercare in a transplantation center” is required, and there is no reason why this should be handled differently in xenotransplantation.

## 5 Conclusion

Consequently, the purported ethical dilemma between the right to withdraw and the need for long‐term monitoring does not exist. Xenotransplant recipients, like all transplant recipients, must remain under continuous medical supervision to preserve transplant function and life. This continuous care inherently provides the framework for long‐term surveillance and early detection of potential infections, thereby protecting both the recipient and the public. Withdrawal from immunosuppressive treatment would be life‐threatening for the patient, but it also means that the patient would not pose a risk to society anymore. As donor screening improves and clinical experience accumulates, evidence will likely continue to show that viral transmission can be minimized or avoided, further enhancing the safety profile of xenotransplantation.

## Author Contributions

J.D. conceived, researched, and wrote the manuscript in its entirety.

## Funding

The author has nothing to report.

## Ethics Statement

Not applicable.

## Consent Statement

No patient or participant consent is required.

## Conflicts of Interest

The author declares no conflicts of interest.

## Data Availability

The authors have nothing to report.

## References

[1] A. S. Iltis , H. J. Silverman , and R. M. Sade , “Research Ethics: Must Subjects Waive the Right to Withdraw from a Xenotransplant Clinical Trial?,” Journal of Thoracic and Cardiovascular Surgery 167, no. 5 (2024): 1880–1884, 10.1016/j.jtcvs.2023.07.007. 37454725

[2] J. W. Entwistle , R. M. Sade , and D. H. Drake , “Clinical Xenotransplantation Seems Close: Ethical Issues Persist,” Artificial Organs 46, no. 6 (2022): 987–994, 10.1111/aor.14255. 35451522

[3] W. J. Hawthorne , “Ethical and Legislative Advances in Xenotransplantation for Clinical Translation: Focusing on Cardiac, Kidney and Islet Cell Xenotransplantation,” Frontiers in Immunology 15 (2024): 1355609, 10.3389/fimmu.2024.1355609. 38384454 PMC10880189

[4] C. Bobier , D. J. Hurst , D. Rodger , and A. Omelianchuk , “Xenograft Recipients and the Right to Withdraw from a Clinical Trial,” Bioethics 38, no. 4 (2024): 308–315, 10.1111/bioe.13262. 38183638

[5] C. A. Bobier , A. Omelianchuk , D. Rodger , and D. Hurst , “Public Health, Xenozoonosis, and the Right to Withdraw from Long Term Xenotransplant Biosurveillance,” The New bioethics: a multidisciplinary journal of biotechnology and the body 31 (2025): 75–85, 10.1080/20502877.2025.2572255. 41082681

[6] X. Hu , D. K. C. Cooper , D. Golshayan , et al., “Xenotransplantation: From Proof of Concept to Clinical Reality,” Swiss Medical Weekly 155 (2025): 4945, 10.57187/s.4945. 41085342

[7] B. P. Griffith , C. E. Goerlich , A. K. Singh , et al., “Genetically Modified Porcine‐to‐Human Cardiac Xenotransplantation,” New England Journal of Medicine 387, no. 1 (2022): 35–44, 10.1056/NEJMoa2201422. 35731912 PMC10361070

[8] B. P. Griffith , A. Grazioli , A. K. Singh , et al., “Transplantation of a Genetically Modified Porcine Heart into a Live human,” Nature Medicine 31, no. 2 (2025): 589–598, 10.1038/s41591-024-03429-1. 39779924

[9] M. M. Mohiuddin , A. K. Singh , L. Scobie , et al., “Graft Dysfunction in Compassionate Use of Genetically Engineered Pig‐to‐Human Cardiac Xenotransplantation: A Case Report,” The Lancet 402, no. 10399 (2023): 397–410, 10.1016/S0140-6736(23)00775-4. PMC10552929 37393920

[10] L. Mou , Z. Pu , and D. K. C. Cooper , “Clinical Xenotransplantation of Gene‐Edited Pig Organs: A Review of Experiments in Living Humans Since 2022,” Medicine Bulletin 1 (2025): 77–85, 10.1002/mdb2.70003.

[11] J. A. Fishman , “Risks of Infectious Disease in Xenotransplantation,” New England Journal of Medicine 387, no. 24 (2022): 2258–2267, 10.1056/NEJMra2207462. 36516091

[12] M. Sekijima , S. Waki , H. Sahara , et al., “Results of Life‐Supporting Galactosyltransferase Knockout Kidneys in Cynomolgus Monkeys Using Two Different Sources of Galactosyltransferase Knockout Swine,” Transplantation 98, no. 4 (2014): 419–426, 10.1097/TP.0000000000000314. 25243512 PMC4174467

[13] K. Yamada , M. Tasaki , M. Sekijima , et al., “Porcine cytomegalovirus Infection Is Associated with Early Rejection of Kidney Grafts in a Pig to Baboon Xenotransplantation Model,” Transplantation 98, no. 4 (2024): 411–418, 10.1097/TP.0000000000000232. PMC4176773 25243511

[14] J. Denner , M. Längin , B. Reichart , et al., “Impact of Porcine cytomegalovirus on Long‐Term Orthotopic Cardiac Xenotransplant Survival,” Scientific Reports 10, no. 1 (2020): 17531, 10.1038/s41598-020-73150-9. 33067513 PMC7568528

[15] H. Jhelum , R. Schäfer , B. B. Kaufer , and J. Denner , “Porcine Cytomegalovirus/Porcine Roseolovirus, Previously Transmitted During Xenotransplantation, Does Not Infect Human 293T and Mouse Cells with Impaired Antiviral Defense,” Viruses 18, no. 1 (2025): 21, 10.3390/v18010021. 41600786 PMC12846669

[16] J. Denner , “How Does a Porcine Herpesvirus, PCMV/PRV, Induce a Xenozoonosis,” International Journal of Molecular Sciences 26, no. 8 (2025): 3542, 10.3390/ijms26083542. 40332048 PMC12026653

[17] R. R. Razonable , “Rare, Unusual, and Less Common Virus Infections after Organ Transplantation,” Current Opinion Organ Transplantation 16, no. 6 (2011): 580–587, 10.1097/MOT.0b013e32834cdaf2. 22001713

[18] C. Bobier , D. Rodger , and D. J. Hurst , “Letter to the Editor. Xenotransplantation and Lifelong Monitoring,” American Journal of Transplantation 24 (2024): 697–698, 10.1016/j.ajt.2023.11.010. 37995839

[19] J. Noordergraaf , A. Schucker , M. Martin , et al., “Pathogen Elimination and Prevention within a Regulated, Designated Pathogen Free, Closed Pig Herd for Long‐Term Breeding and Production of Xenotransplantation Materials,” Xenotransplantation 25 (2018): e12428, 10.1111/xen.12428. 30264879 PMC7169735

[20] L. S. Gazda , J. Collins , A. Lovatt , et al., “A Comprehensive Microbiological Safety Approach for Agarose Encapsulated Porcine Islets Intended for Clinical Trials,” Xenotransplantation 23 (2016): 444–463, 10.1111/xen.12277. 27862363 PMC7169751

[21] J. Denner , “Virus Safety of Xenotransplantation,” Viruses 14, no. 9 (2022): 1926, 10.3390/v14091926. 36146732 PMC9503113

[22] H. Jhelum , B. B. Kaufer , and J. Denner , “Comprehensive Protocols for Detecting Xenotransplantation‐Relevant Viruses,” Methods and Protocols 8, no. 5 (2025): 109, 10.3390/mps8050109. 40981227 PMC12452624

[23] E. Plotzki , M. Keller , D. Ivanusic , and J. Denner , “A New Western Blot Assay for the Detection of Porcine cytomegalovirus (PCMV),” Journal of Immunological Methods 437 (2016): 37–42, 10.1016/j.jim.2016.08.001. 27498035

[24] S. Halecker , S. Hansen , L. Krabben , F. Ebner , B. Kaufer , and J. Denner , “How, Where and When to Screen for Porcine cytomegalovirus (PCMV) in Donor Pigs for Xenotransplantation,” Scientific Reports 12, no. 1 (2022): 21545, 10.1038/s41598-022-25624-1. 36513687 PMC9747970

[25] “Permissible Medical Experiments,” in Trials of War Criminals Before the Nuremberg Military Tribunals Under Control Council Law No 10, Nuremberg (U.S. Government Printing Office, 1946), 181–182.

[26] Department of Health and Human Services (HHS) , Guidance on Withdrawal of Subjects from Research from the Department of Health and Human Services (HHS), 2010, https://www.hhs.gov/ohrp/sites/default/files/ohrp/policy/subjectwithdrawal.pdf.

[27] Food and Drug Administration (FDA) , Source Animal Product, Preclinical, and Clinical Issues concerning the Use of Xenotransplantation Products in Humans. Guidance for Industry, 2016, https://www.fda.gov/media/102126/download.

[28] M. A. Spillman and R. M. Sade , “Clinical Trials of Xenotransplantation: Waiver of the Right to Withdraw from a Clinical Trial Should be Required,” The Journal of Law, Medicine and Ethics 35, no. 2 (2007): 265–272, 10.1111/j.1748-720X.2007.00135.x. 17518852

[29] R. M. Sade and R. Mukherjee , “Ethical Issues in Xenotransplantation: The First Pig‐to‐Human Heart Transplant,” The Annals of Thoracic Surgery 113, no. 3 (2022): 712–714, 10.1016/j.athoracsur.2022.01.006. 35168784

[30] M. Rothblatt , “Commentary on Achievement of First Life‐saving Xeno‐heart Transplant,” Xenotransplantation 29 (2022): e12746, 10.1111/xen.12746. 35471736

[31] Homer , The Odyssey (Oxford World's Classics) (Oxford University Press, 2008).

[32] D. J. Hurst , L. Padilla , T. Schiff , and B. Parent , “Revisiting the Use of Ulysses Contracts in Xenotransplantation,” Transplantation 108, no. 2 (2024): 369–373, 10.1097/TP.0000000000004679. 37246302

